# TEM Microstructure, Mechanical Properties and Temperature Estimation in the 5XXX Series Al-Mg-Si Aluminum Alloy with W-Ni-Fe Tungsten Composite Friction-Welded Joints

**DOI:** 10.3390/ma15031162

**Published:** 2022-02-02

**Authors:** Radosław Winiczenko, Mieczysław Kaczorowski, Anna Krzyńska, Olgierd Goroch, Andrzej Skibicki, Paweł Skoczylas

**Affiliations:** 1 Institute of Mechanical Engineering, Warsaw University of Life Sciences, 02-787 Warsaw, Poland; 2 Institute of Mechanics and Printing, Warsaw University of Technology, 02-524 Warsaw, Poland; m.kaczorowski@wip.pw.edu.pl (M.K.); o.goroch@wip.pw.edu.pl (O.G.); p.skoczylas@wip.pw.edu.pl (P.S.); 3 Institute of Manufacturing Technologies, Warsaw University of Technology, 02-524 Warsaw, Poland; a.krzynska@wip.pw.edu.pl; 4 Faculty of Mechanical Engineering, Bydgoszcz University of Science and Technology, 85-789 Bydgoszcz, Poland; askibic@utp.edu.pl

**Keywords:** friction welding, W-Ni-Fe composite, TEM, temperature distribution

## Abstract

The temperature distributions, microstructure, and mechanical properties of tungsten composite with aluminum alloy friction-welded joints are presented in this paper. The effects of welding parameters on flash diameter, shortening, joint efficiency, microhardness, and microstructure were studied. Empirical temperature models for heating and cooling phases are proposed in this study. The predicted maximum temperatures at the periphery and in the axis of aluminum specimens were close to 550 °C and 480 °C at the interface, respectively. Moreover, the peak temperature in the weld zone was studied analytically. A maximum tensile strength of 234 MPa was reached for the following welding parameters: friction time of 3.5 s and friction force of 12.5 kN. The efficiency of the welded samples decreased after reaching the maximum value, with an increase of friction time and force. Maximum hardness at the interface and the half-radius reached 100 HV and 80 HV in the aluminum alloy joints, respectively. Dynamic recrystallisation areas on the aluminum alloy side were observed. Transmission electron microscopy observations of the microstructure in the aluminum alloy revealed the presence of a high dislocation density compared to the parent material.

## 1. Introduction

Rotary friction welding (FRW) is a solid-state welding method in which coalescence of the joining surface is achieved without melting. FRW uses heat generated from the friction between a moving workpiece and a stationary one to produce the weld. In continuous direct drive friction welding (CDFW), one of the workpieces driven by a motor is rotated continuously at a predetermined speed, while the other is restrained from rotation. Heat is generated as the faying surfaces rub together under pressure. The rotational driving force is terminated at a preset time, and the rotating workpiece is stopped by applying a brake force or its resistance to rotation. The upsetting pressure must be maintained or increased after rotation ceases to produce a sound weld. In inertia friction welding (IFW), one of the workpieces is connected to a flywheel, and the other is restrained from rotation. The stored energy in the flywheel, decelerated from a preset speed, causes the two faying surfaces to rub together under pressure and produce a weld [1].

Welding parameters such as friction force (FF) and upsetting pressure (UP), rotational speed (RS), and welding time (WT) play a major role in determining the joint quality [2]. The rotational speed and applied pressure affect the shape of the welded zone and the width of the heat affected zone (HAZ) [3]. The application of high pressure produces a narrow HAZ, while using a high rotating velocity causes an extension of the HAZ and enlarged grain size. For the IFW, the controlling welding parameters are initial rotating velocity, moment of inertia, velocity of rotary part at termination of process, and axial pressure. In the case of CDFW, the process parameters are rotational speed, initial pressure, secondary pressure, duration of forging stage, and total time of friction welding [4].

FRW is a solid-state technique, successfully applied to join many engineering materials having different physical and mechanical properties, such as ceramic [5], plastic/polymer materials [6], metal matrix cast composite [7,8], tungsten heavy alloy [9], or ductile iron [10,11,12].

Tungsten composite alloys (TCAs) are economically fabricated using a powder metallurgy (PM) technique, including conventional liquid phase sintering (LPS) and infiltration. These composite materials contain nearly spherical tungsten grains embedded in the matrix phase, Ni-base solid solution, containing elements such as W, Fe, Co, Cu, Mn, and other elements [13]. TCAs have a high strength and density, excellent corrosion resistance, relatively high thermal conductivity, and low coefficient of thermal expansion. The mechanical properties of TCAs depend strongly on the particle size and purity of the selected powders [14]. These unique properties make them attractive materials for many engineering applications, such as radiation shielding, counterweights, inertial masses, and kinetic energy penetrators (KEPs) [15]. KEPs are equipped with an aluminum alloy ballistic cup that protects the projectile against ricochets when it hits the armor plate. The nose of the armor is often covered with a ballistic cap because of splashing. This cap should be made of soft metal. Its task is to weaken the adverse phenomena occurring when hitting the armor.

The ballistic cups can be joined to the tungsten composite part of the projectile by a thread. However, this is a complicated and expensive process. Tungsten heavy alloy is often joined by conventional fusion welding methods, such as diffusion bonding [16,17,18], brazing [19,20], or electron beam welding [21,22]. Problems include cracks, brittle intermetallic phase formation, weld porosity, and grain growth due to the increased temperature in fusion welding. A more efficient joining method might be the (FRW) method.

The main goal of this study was to verify the possibility of using FRW as a method of ensuring a high-strength joint between a tungsten heavy-alloy core and soft aluminum ballistic nose. The effect of friction force and friction time on the joint efficiency and hardness distributions of samples were investigated. The morphology, microstructure, and mechanical properties of W-Ni-Fe composite with 5XXX series Al-Mg-Si aluminum alloy joints were examined. Additionally, the temperature distribution of the welded joints was measured and compared with a numerical model.

## 2. Materials and Methods

### 2.1. Materials Selection

A standard 5XXX series Al-Mg-Si aluminum alloy (in annealed condition) and conventional W-Ni-Fe tungsten composite with typical 7:3 nickel to iron ratio were used for the welding process. The main components of the aluminum alloy were magnesium and manganese (see Table 1). Tungsten composite (TC) was prepared using the powder metallurgy (PM) method. TC was fabricated by mixing an appropriate amount of powder, compacting, and then liquid-phase sintering in a hydrogen atmosphere, as noted in previous papers [23,24].

**Figure 1:**
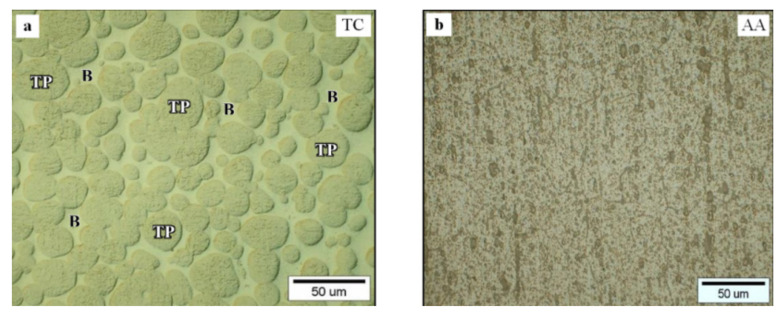
Microstructures of base materials: (**a**) tungsten composite, (**b**) aluminum alloy.

Figure 1a shows the tungsten spherical particles (TP) surrounded by a solid solution matrix (Ni-Fe) as the binder phases (B). The microstructure of aluminum alloy (AA) consists of many undissolved second-phase intermetallic particles (see Figure 1b).

Mechanical properties such as tensile strength (TS), 0.2% yield strength (YS), elongation EL (%), hardness (HB), and chemical composition of materials are shown in Table 1.

### 2.2. Friction Welding Setup

**Figure 2:**
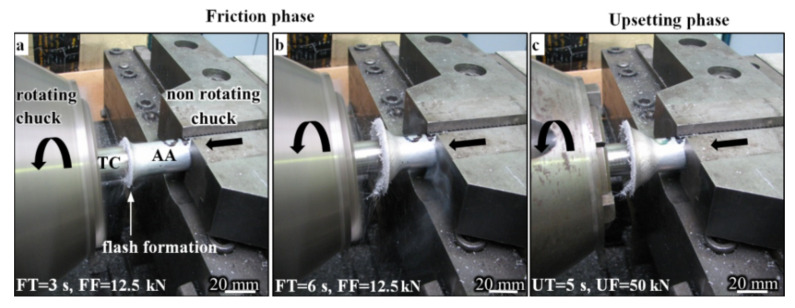
Continuous drive-friction welding process: (**a**,**b**) friction phase, (**c**) upsetting phase.

Samples with a diameter of 20 mm and a length of 100 mm were prepared for the welding process. A friction welding experiment was conducted using a drive-friction welding machine (ZT-13, ASPA, Wrocław, Poland), as shown in Figure 2.

The friction welding process was divided into the friction phase (Figure 2a,b) and the upsetting phase (Figure 2c). TC samples were placed in the spindle, while the samples from the aluminum alloy were supported by the welding machine. The friction process took place after the axial pressure on the samples. The friction time lasted from 0.5 to 9.5 s. During this phase, the AA sample was shortened, and the flash formed slowly. The second phase of the friction welding process was the upsetting phase, which lasted for five seconds. During this phase, the spindle stopped abruptly. Then the axial pressure was exerted on the samples [25]. The result of this process was the formation of a flash on the AA part, as shown in Figure 2c.

Table 2 presents the range of welding parameters, where the rotational speed of 1450 rpm, the upsetting force of 50 kN, and upsetting time of 5 s were constant values.

In this study, only the relationship between friction force and friction time was considered using the experimental planning method. The upsetting time did not have a significant impact on joint strength efficiency, as shown in the references [26,27].

### 2.3. Methods

#### 2.3.1. Tensile Test

A tensile test was performed for each variant of welding parameters (see Table 2) for three repetitions. The tensile tests were performed on three samples according to PN-EN ISO 10002-1 standard. The tensile test was performed on a Instron (Norwood, MA, USA, 115-PFZ100) type universal machine.

#### 2.3.2. The Microhardness Test

A Vickers microhardness test was carried out on a Zwick machine (Zwick Roell, Ulm, Germany). A load of 100 g and a dwell time of 15 s were applied. Hardness was tested according to the PN-EN ISO 6507-1:2018-05 standard. Three specimens were used for measurements.

#### 2.3.3. The Temperature Measurements

**Figure 3:**
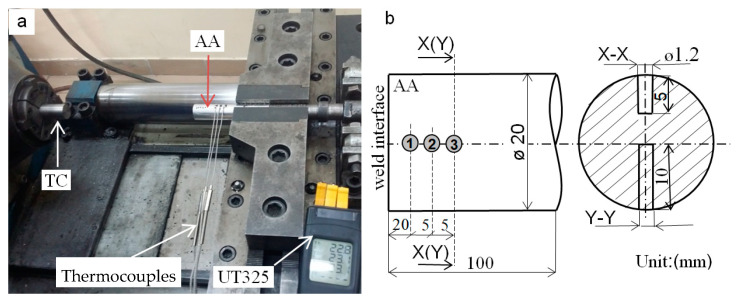
(**a**) Experimental setup of thermocouples in the nonrotating aluminum sample, and (**b**) a schematic representation of the arrangement.

A TP203K1b2001 type thermocouple (Czaki Thermo-product, Raszyn, Poland) with an accuracy of ± 0.1 °C was used for the temperature measurements. The thermocouples were inserted into drilled holes (see Figure 3b) with a diameter of 1.2 mm, made at different distances from the weld interface.

Thermocouples were placed in both the axis center and at 1/2 of the sample radius in the aluminum sample. During the welding process, an automatic temperature measurement was made using the UT325 LCD digital recorder (Uni-Trend Technology, Dongguan City, China) with a frequency of 1000 Hz.

#### 2.3.4. TEM Preparation

Samples of 0.1 mm thickness were sliced from the 3 mm diameter rod with an axis positioned 3 mm from the center, being parallel to the axis of the joined aluminum rod. Three successive slices were cut using a load-less wire saw from the aluminum part, located in the vicinity, at a distance of 10 and 20 mm from the weld interface. Then, the specimens were electro-polished using a two-jet method. Thin foils were observed using a high-voltage TEM (JEM-3010, JEOL Ltd., Tokyo, Japan) with a 300 kV accelerating voltage.

## 3. Results

### 3.1. Effect of Plastic Deformation

The friction welding force mostly affects the material that has the lower hardness. In this case, it was the aluminum alloy sample.

**Figure 4:**
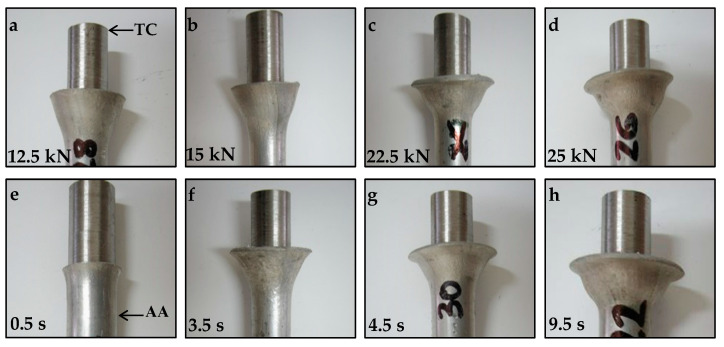
The appearance of the TC/AA joints at various friction forces and at a constant friction time equal to 9.5 s (**a**–**d**), and the influence of various friction times at a constant friction force equal to 12 kN (**e**–**h**).

Figure 4 shows the appearance of the TC/AA joints at various friction forces (Figure 4a–d) and friction times (Figure 4e–h).

The extensive shortening of the aluminum alloy specimens can be seen from Figure 4c,d,g,h. Shortening of the specimen is not desirable, but it depends on the friction welding parameters, especially on friction force and friction time. The shortening is caused by the fact that the temperature at the weld interface is close to the melting point of 5XXX, which results in a considerable decrease of hardness properties, enabling a very intensive plastic deformation. A significant increase in flashes can be seen with the increase of friction force (see Figure 4c,d) and welding time (see Figure 4g,h).

**Figure 5:**
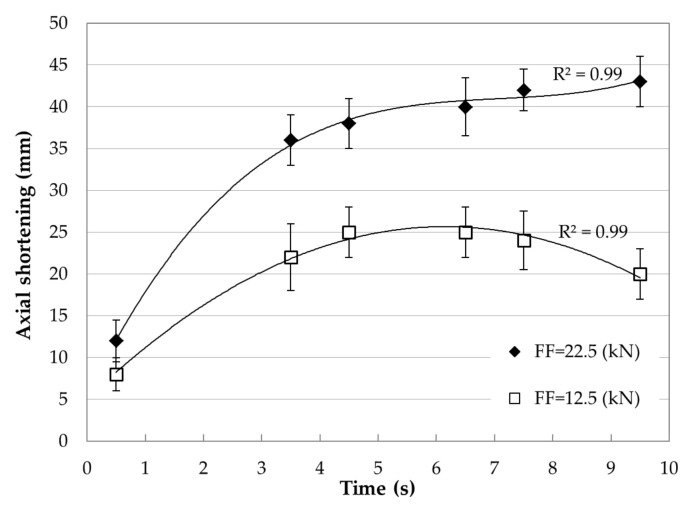
Effect of welding parameters on the axial shortening of friction-welded aluminum alloy with tungsten composite.

Figure 5 shows the influence of welding time and friction force on the axial shortening of the AA/TC welded joint.

It can be seen that as the friction force and welding time increased, the axial shortening of the aluminum samples increased.

During friction welding of dissimilar materials, a narrow diffusion zone on both sides of the joint may occur, as well as the precipitation of intermetallic phases and significant plastic deformation of the welds [10,15].

### 3.2. Effect of Welding Parameters on Joint Efficiency

**Figure 6:**
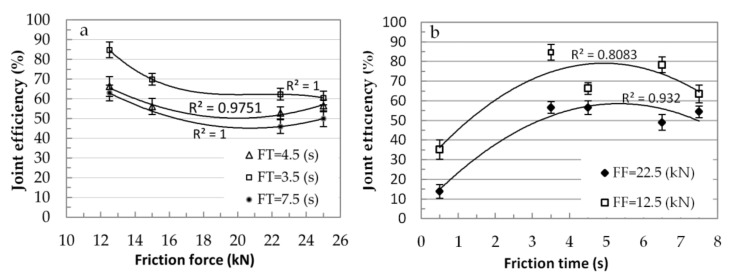
Effect of friction welding parameters on joint efficiency; (**a**) friction force vs joint efficiency, (**b**) friction time vs joint efficiency.

Figure 6 shows the relationship between the welding parameters and joint efficiency of the welded joint.

The joint efficiency (JE) was the ratio between the tensile strength (TS) of the welded joint and the ultimate tensile strength (UTS) of the base metal, as proposed in paper [28]. The effect of friction force on the joint efficiency is shown in Figure 6a. It can be seen from the diagram that the joint efficiency decreased with an increasing friction force. The graph in Figure 6a shows that the increase of friction force to 20 kN caused a decrease of JE with a constant friction time. Then, the JE slightly increased with increasing friction force, and reached about 50 and 65% for friction time of 7.5 and 3.5 s, respectively. Figure 6b shows the effect of friction time on joint efficiency. The JE increased with increasing friction time, and it was approximately 85% at a friction time of 3.5 s with a friction force of 12.5 kN. Next, the joint efficiently slightly decreased with increasing friction time, and it was about 60% and 50% for friction forces of 12.5 and 22.5 kN, respectively.

### 3.3. The Vickers Hardness Distributions

**Figure 7:**
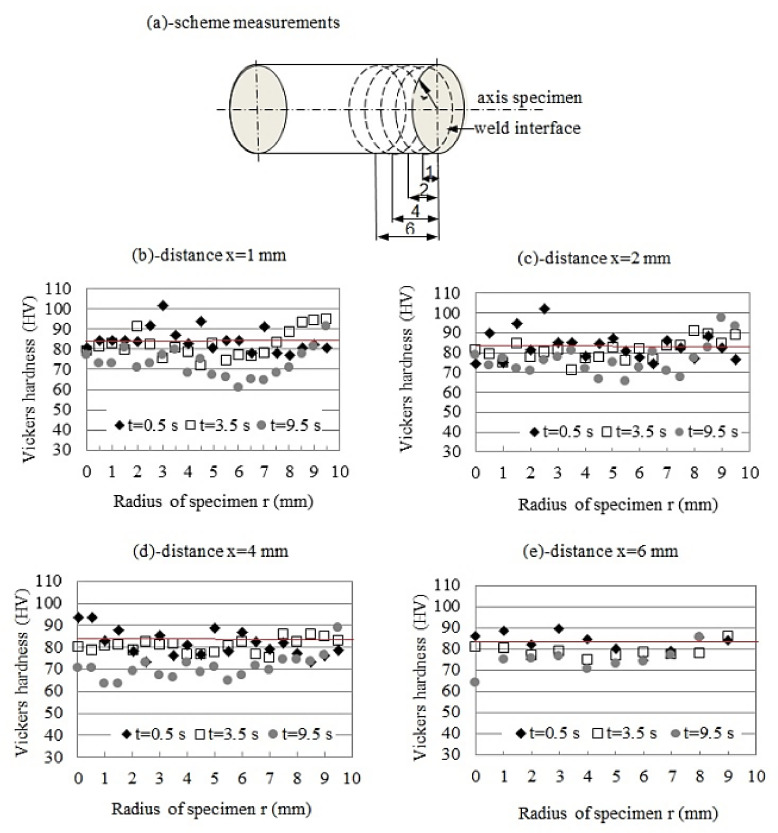
Effect of friction time on the microhardness profiles as a function of distance from the specimen axis-r at different distances from the interface-x.

The above results show that the hardness of the TC/AA joint depends on the welding parameters. The highest hardness can be achieved at a friction force of 12.5 kN, at a friction time of 3.5 s. The Vickers hardness distributions in the aluminum alloy samples are depicted in Figure 7. These are the average of three measurements.

In the graphs (Figure 7b–e), the hardness distributions of the aluminum alloy are shown as a function of distance: 1, 2, 4, and 6 mm from the interface, measured according to the scheme in Figure 7a.

Figure 7b–d illustrates the change in hardness of aluminum alloy welded to tungsten composite using the following parameters: friction force 12.5 kN, and friction times of 0.5, 3.5, and 9.5 s, respectively. As can be seen, in most cases, the hardness distribution had a complex character. The most homogeneous character of hardness changes, depending on the distance from the axis of the specimen and the joint interface, was observed at a friction force of 12.5 kN and friction time of 3.5 s. Such a time enabled achieving the maximal value of tensile strength, equal to 234 MPa.

### 3.4. Estimation of Temperature Using an Analytical Model and an Experiment

The friction welding process heats the materials that are being joined. As the rotational velocity is the highest in the peripheral regions of specimens being joined, the heat is generated in the first place in those areas. Then it is transferred in the direction of their axes, as reported by the authors of [27,28]. Heating the joined materials is accompanied by cooling external surfaces, during the friction stage and after its completion. Therefore, it should be expected that the heating volume of the joined materials lying at various distances from the specimen’s axes and the weld interface will be different. Considering the temperature close to the joint, which should not exceed a value of 650 °C, only aluminum alloy was analyzed. The friction welding process is a solid-state joining method. Therefore the joining is performed at a temperature below the melting point of the materials [24].

Based on a one-dimensional transient-heat conduction analysis, the temperature (*T*) at different distances (*x*) from the interface as a function of time (*t*) is:

(1) $$T\left(x,t\right)=T_{0}+\frac{2q_{0}\sqrt{\frac{at}{\pi }}}{\lambda }exp\left(-\frac{x^{2}}{4at}\right)-\frac{q_{0}x}{\lambda }\left(1-erf\left(\frac{x}{2\sqrt{at}}\right)\right)$$

where *T*_0_, *q*_0_, α, λ, and *erf* are the room temperature, heat input, thermal diffusivity, thermal conductivity, and error function, respectively. Thermal diffusivity is normally expressed as:

(2) $$a=\frac{\lambda }{\rho \;c_{p}}$$

where *ρ*, *c_p_* are the density and heat capacity. The values *ρ*, *c_p_*, and *λ* are 2.69 (g/cm^3^), 900 J/(kg·K), and 134 (W/m·K) for AA5054, respectively (www.matweb.com, accessed on 31 December 2021). Therefore, the thermal diffusivity was calculated to be 5.53 × 10^−4^ m^2^/s.

(3) $$q_{0}=\frac{2}{3}\mu P_{n}\omega r$$

where *μ*, *P_n_*, *ω*, and *r* are the average friction coefficient, normal pressure, angle velocity, and radius of the specimen, respectively. The values for *P_n_*, *ω*, and *r* are 40 MPa, 152 r/s, and 10 mm, respectively. To ensure the accuracy of Equation (1), initially, the temperature calculated by Equation (1) after 9.5 s was compared. The friction coefficient of 0.12 for aluminum alloy was used, according to reference [25]. This value provided the best possible agreement between the computed and experimental temperatures.

### 3.5. Temperature Distributions

Friction welding can be divided into rapid heating and continuous cooling processes. A rapid temperature rise occurs during the heating process due to the intense friction between the samples [26,27].

**Figure 8:**
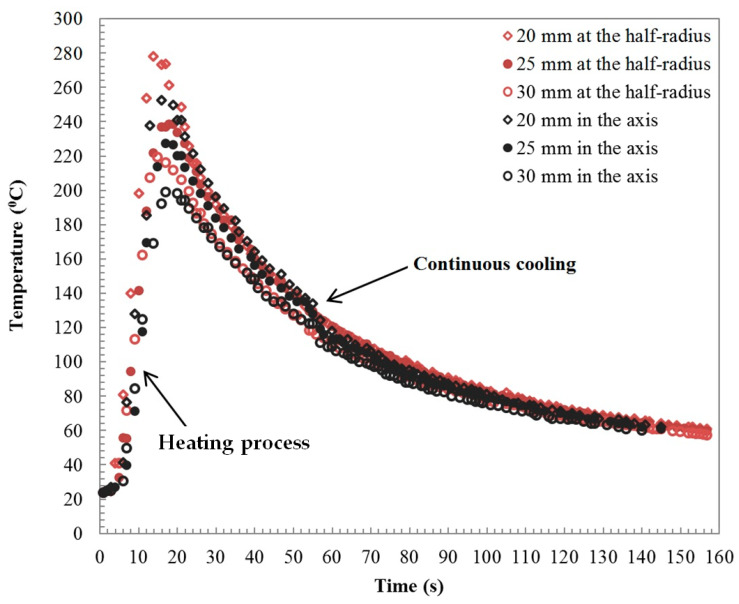
Comparison of time–temperature profiles in the tungsten composite/aluminum alloy friction-welded samples.

Time-temperature profiles at the half-radius and center line of the specimens are shown in Figure 8.

The curves show that the temperature increased immediately after the first phase of the welding process, which was supported by a higher coefficient of friction and plastic deformation, as reported in [28,29]. The peak heating temperature at the half-radius and in the specimen axis equaled 278 and 252 °C, at the distance x = 20 mm from the weld interface (see Figure 8), recorded for a cooling time t = 14 s. At 15 s, the temperature dropped gradually to 80 °C after 85 s. The temperature value did not reach the melting point of aluminum alloy, i.e., 650 °C. The temperature recorded at the half-radius is higher than that at the axis of the specimen. The heating rate (HR) at the half-radius was higher than at the center line of the specimens. The HR of the welding process was calculated as being around 18 and 14 °C/s at the half radius and center of the specimen, respectively.

Figure 8 shows the continuous cooling process after the upsetting phase of the welding process. The temperature profiles in the axis and half-radius of the specimen clearly show that the temperature at the half-radius was higher than that at the center line. The temperature profiles during continuous cooling have a similar character below a temperature of approximately 80 °C. The cooling rate CR = 1.3 °C/s in the axis of the specimen was relatively slow compared to the HR = 14 °C/s.

### 3.6. Mathematical Modelling of Temperature

Temperature models of the welding process at half-radius (Table 3) and the axis of specimens (Table 4) are presented.

The constant models such as *a*, *b*, *c*, and the coefficient of determination *R*^2^ were calculated using the genetic algorithm approach.

Genetic algorithms are optimization methods using the process of evolution in the natural environment [30]. A genetic algorithm assesses a single individual (chromosome) based on fitness function in a population. The scheme of the genetic algorithm procedure was presented in the work in [29].

The fitness functions were the empirical models for heating:

(4) $$T=\frac{a}{b+exp\left(c\cdot t\right)}$$

and

(5) $$T=a-b\;\cdot t^{c}$$

cooling processes, proposed in paper [29].

The operators of the genetic algorithm are crossover and mutation [31]. A population size of 60, the number of generations of 200, the rates of crossover of 0.8, and the mutation of 0.075 were applied in this study. The simulation process was carried out in MATLAB 7.6 R2008a software, 2008 (MathWorks, Portola Valley, CA, USA) [32].

**Figure 9:**
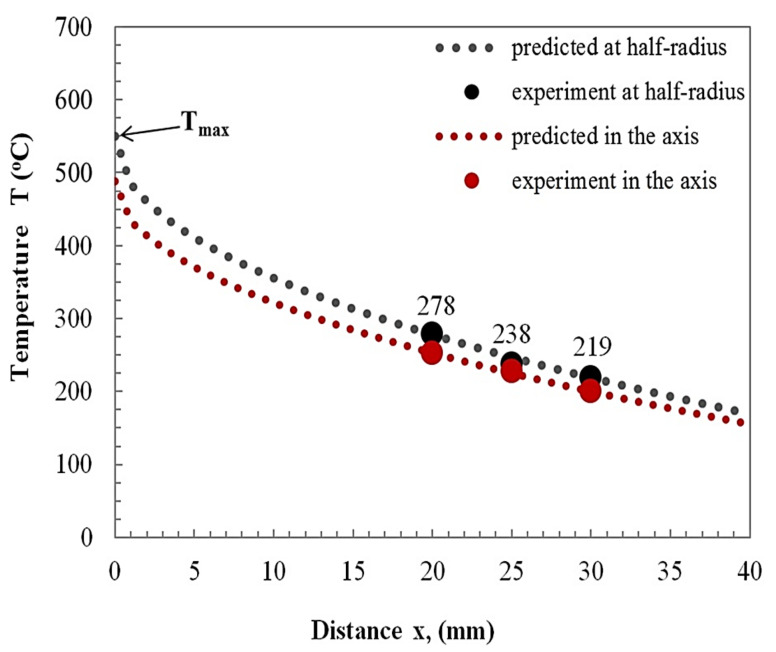
Comparison of the predicted maximum temperature on aluminum alloy side at the weld interface.

Figure 9 shows the maximum temperatures predicted using a genetic algorithm as a function of distance from the weld interface.

The peak temperatures were estimated using the empirical models:

(6) $$T_{max}=550-63.77\cdot x^{0.484}$$

and

(7) $$T_{max}=488-52.64\cdot x^{0.5}$$

where *x* is the distance from the weld interface (see Table 5).

The empirical models’ goodness fit the examined data, with a higher R-squared = 0.99. The predicted maximum temperatures in the axis and half-radius of the specimen amounted to 488 and 550 °C, respectively. The maximum temperature of aluminum alloy (550 °C) was lower by about 100 °C than the melting temperature of the base material (~650 °C).

### 3.7. Microstructure Observation in TEM

TEM tests were carried out with aluminum, which underwent a significant plastic deformation (see Figure 4). The microstructure observations were conducted for the base material and as a function of a 0.1, 10 and 20 mm distance from the weld interface.

**Figure 10:**
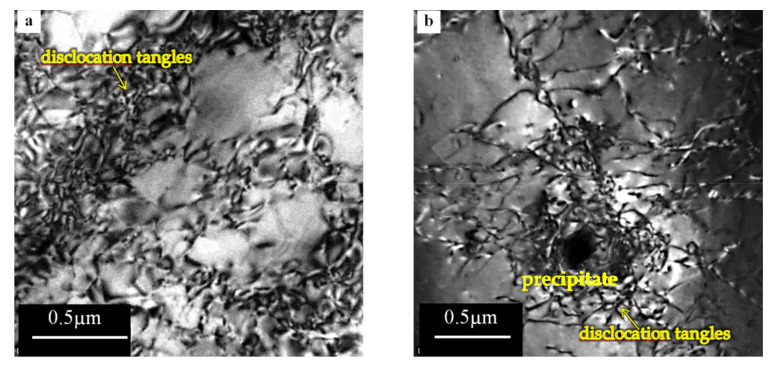
TEM microstructure of the base aluminum alloy: (**a**) dislocations are not uniform distributed, (**b**) dislocation tangles blocked at the large precipitate.

The microstructure of aluminum alloy base material is shown in Figure 10.

The micrograph (Figure 10a) shows that the dislocations are not uniformly distributed. There is an area of high density and areas devoid of dislocations. Moreover, it can be seen that the dislocations form weakly defined sub-cell microstructures (Figure 10a). Figure 10b shows the dislocation tangles blocked at the large precipitate.

**Figure 11:**
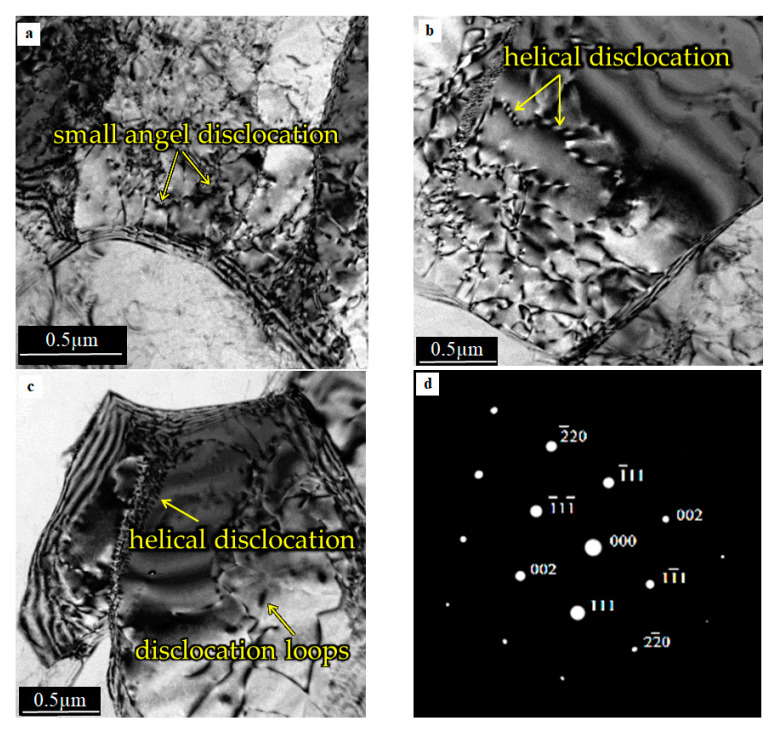
TEM microstructure in the aluminum specimen laying in the immediate vicinity of the weld interface: (**a**–**c**) relatively small grains divided with a high angle the grain boundaries, (**d**) selected diffraction pattern (SAD) from dark grain is shown in Figure 11c (zone axis [110]).

Figure 11 shows the microstructure at the immediate vicinity of the weld interface. The micrographs reveal the presence of a relatively high dislocation density, as compared to the base material (Figure 10).

The small grains are divided with high-angle grain boundaries. Dislocation loops and precipitates formed on dislocations (Figure 11a,c). A moderate density of helical dislocation, marked by arrows, is observable in Figure 11b. A similar dislocation microstructure in the friction stir welded aluminum alloy can be found in [33]. The almost perfect symmetry of the diffraction spots proves that the network is not distorted, as shown in Figure 11d. The above suggests that the grains observed in the samples resulted from the recrystallisation process. This process took place under the influence of the heat generated during friction welding, as reported in [26].

**Figure 12:**
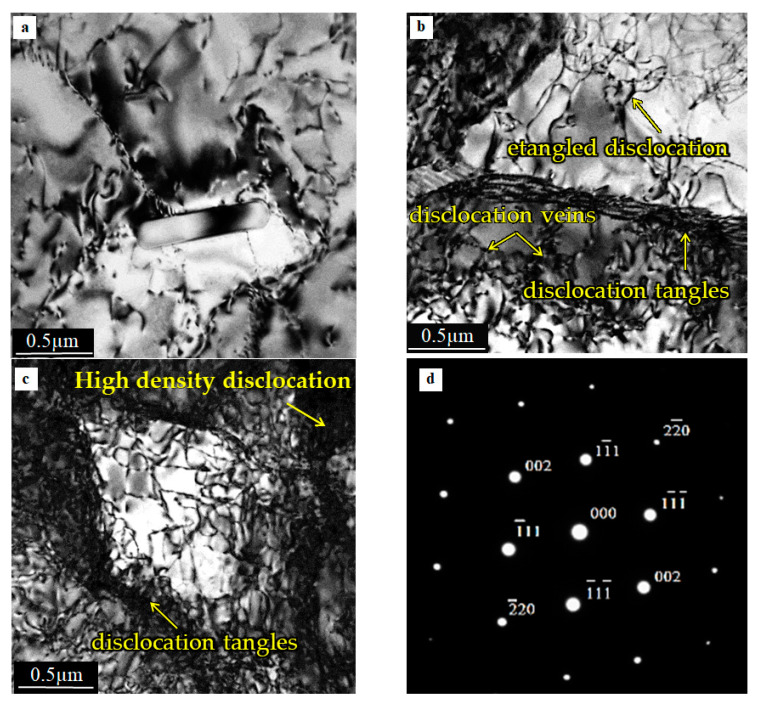
Micrograph showing microstructure in aluminum alloy friction weld at a distance 10 mm from the interface (**a**–**c**), (**d**) SAD from dark grain showed in Figure 12b.

The microstructure of AA, located 10 mm from the TC/AA weld interface is shown in Figure 12.

Small precipitates are observable at the central part of the micrograph. Additionally, small-angle dislocation grain sub-boundaries are visible in the micrograph. A similar distribution of dislocation is observed in Figure 12b. However, as follows from the difference in contrast, the up and down grains visible in the micrograph are divided by the high angle boundaries. Figure 12b,c shows that some simple dislocation patterns, such as dislocation veins and tangles, can still be found in the grain and at the grain boundary. Similar observations of the dislocation microstructure were reported by authors [34]. The micrograph depicted in Figure 12c shows a distorted region with a high dislocation density accumulated in the sub-grain boundary. It can be seen that, despite a high dislocation density, there is no sign of distortion in the selected diffraction pattern SAD photo, Figure 12d.

**Figure 13:**
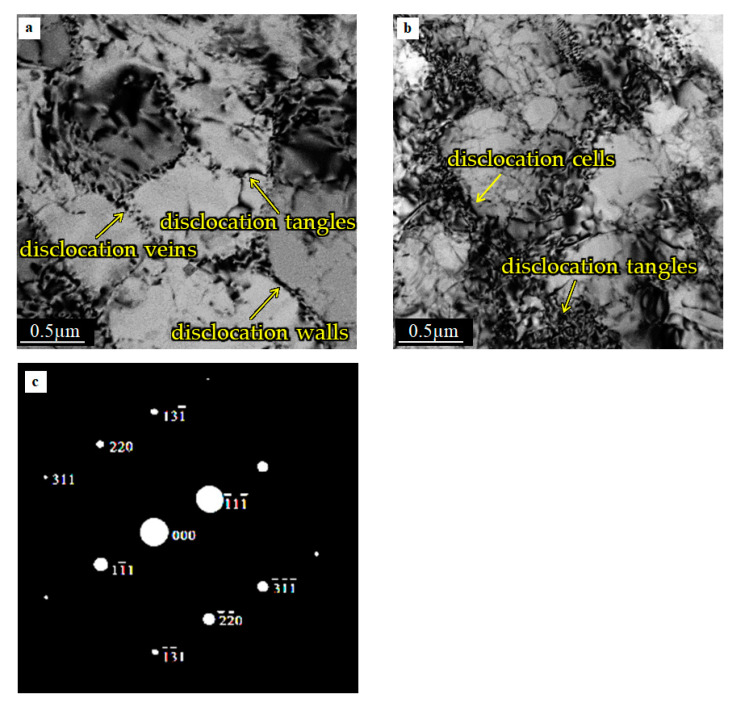
The microstructure in the aluminum part of the weld at the distance of 20 mm from the interface (**a**,**b**), SAD from the sub-grains arranged in the central part of Figure 13b (**c**).

The micrograph presented in Figure 13 shows the aluminum alloy microstructure on the side located about 20 mm from the weld interface.

In two-electron micrographs (Figure 13a,b), typical dislocation cell microstructures are visible. The mean diameter of the dislocation cell is approximately 2–3 µm, and often even less. Dislocation walls and incipient cells prevailed, and dislocation veins (see Figure 13a) are observed in the interior regions. The dense dislocation tangles tend to form walls in the grain (Figure 13b). There is no evidence of marks such as diffusivity, stretching, or curvature of the diffraction spots on diffraction pattern (see Figure 13c). It can be seen that the microstructure showed in Figure 13 is very similar to the base material presented in Figure 10.

Very coarse grains in the zone most adjacent to the joint interface were observed during the microscopic observation, as reported in a previous study [27,28]. Inside the grains, a small number of dislocations were introduced during the upsetting stage of the welding process [33,34]. According to the authors of [35,36], the dynamic recrystallisation process (DRX) starts during this stage. Therefore, a large amount of shear strain and heat are introduced during the friction phase. Subsequently, there is a large amount of deformation during the upsetting process with high temperature-induced DRX. The aluminum samples were hot worked during the friction phase, and dynamic recrystallisation started under the upsetting pressure. Generally, refined and DRX grains are formed in soft aluminum alloy close to the weld interface [27]. Next, a healing process was observed that was characterized by the arrangement of the dislocation microstructure in the more deeply placed material parts. Consequently, cellular dislocation microstructures, stimulated by seeking for a state of lower thermodynamically free energy, are formed [35,37]. The result of this is a microstructure in which the grains are divided into sub-grains separated by low-angle boundaries. Similar observations were presented by the authors in [37,38].

## 4. Discussion

To begin, it should be explained why only the aluminum part joined with the tungsten composite alloy was tested. First of all, the process of joining is a thermoplastic treatment. The heat generated during the welding process increases temperature, influencing the softening of both joined materials. Deformation scale and the processes accompanying it depend on the melting point of the joined materials. The melting points of aluminum alloy and the Ni-based matrix tungsten composite alloy are 650 °C (923 K) and 1455 °C (1728 K), respectively (www.matweb.com, accessed on 31 December 2021). Considering that the Ni-based matrix is not pure Ni but a solid solution containing W and Fe, its melting point is about 1495 °C (1768 K). Assuming that the homologous temperature is 0.4·Tm, then in the case of aluminum alloy, it equals 370 K (97 °C) and Ni-base matrix 690 K (420 °C). A comparison of these values suggests that the effect of a thermo-mechanical ‘heat treatment’ on the processes and microstructure of the tungsten composite alloy part of the joined part would be negligible compared to the 5XXX alloy part. This was confirmed by the photos in Figure 4e, where no deformation of the tungsten part of the joining materials is visible. It is interesting that the distribution of axial shortening of the aluminum part is a function of the friction time for the two friction forces. It was evident that the axis shortness increased with friction time and friction force. As can be seen from the graphs (Figure 5), the axis shortness was almost twice as significant for a friction force of 22.5 kN than of 12.5 kN.

The graphical results of joining efficiency (JE) as a function of friction forces (Figure 6a) and friction time (Figure 6b) are interesting. Figure 6a shows that the best joining efficiency was obtained with lower friction forces and friction times, regardless of friction time. A confirmation of the former conclusion is found in Figure 6b, wherein with a friction force FF = 12.5 kN for friction times between 4 and 5 s, the JE value reached 75% and is 20% higher compared to a FF = 25 kN.

The graphs presented in Figure 7 illustrate the hardness distribution in aluminum as a function of distance: x = 1, 2, 4, and 6 mm from the interface (see scheme on Figure 7a) for three friction times, 0.5, 3.5, and 9.5 s and constant friction force of 12.5 kN. Among the many graphs obtained for different friction forces in the experiment, a friction time of 12.5 kN was chosen because it produced the best joining strength, achieving 234 MPa. As seen in Figure 7, the hardness distribution had a complex character. The hardness of the parent aluminum alloy rod was 83 µHV, but at welding, the interface achieved 95 µHV, depending on friction time. The average joint hardness decreased as the friction time increased. A softened region can be seen at distance of about 4 to 8 mm from the interface. The highest hardness, of 110 µHV, of the sample was recorded in the joint axis near the weld interface. The hardness in the half-radius specimen slightly decreased to 78 µHV and 65 µHV for FT = 0.5 and 9.5 s, respectively (see Figure 7a,b). The hardness of aluminum alloy at the center was higher than in other areas of the specimen with shorter friction times of 0.5 and 3.5 s. The hardness of the specimen subjected to a longer friction time, e.g., 9.5 s, was lower than that of the base alloy. The surface hardness of the specimen increased and reached the value of parent metal. Finally, the average hardness decreased with the distance from the joint interface. The last graph (Figure 7e) shows no significant changes in the hardness distribution in the plane located 6 mm from the interface.

The hardness changes reflect the microstructure evolution occurring during the rotary friction welding process, including two stages. The first is the heat generated at the interface during friction under loading, causing a continuous temperature increase. The resulting heat is transferred from the interface towards the joining materials, to the region more or less distant from the interface, depending on the friction time and thermal parameters (thermal diffusivity, thermal conductivity, specific heat). The second stage includes the processes occurring during upsetting, when the upsetting force is applied to the material for a given time. In the case of our experiment, both materials were for a time of 5 s subjected to an upsetting force 50 kN. As was stated above, the influence of temperature increase on tungsten composite alloy is negligible; therefore, the research study concentrated on the processes which proceeded in the aluminum specimen. Depending on temperature and time, these could have been grain growth, dynamic recrystallisation, and recovery. Due to the relatively short time of heat impact, the two last processes are the most probable. Recovery usually leads to the restoration of plasticity, while maintaining high strength properties. The recrystallisation process proceeds at the temperature range of 0.4–0.6 Tm, depending on the draft value, consisting of a nucleation end growth of new grains almost free of dislocations accompanied by a decrease of tensile properties.

The micrographs (Figure 11, Figure 12 and Figure 13) show the differences between the microstructure and dislocation distributions in the aluminum alloy joints at various distances from the weld interface. Compared to the microstructure of the starting 5XXX alloy material (Figure 10), where dislocations formed a weakly defined dislocation cell microstructure, only the microstructure at the vicinity of the joining plane is different (Figure 11). There is no question that in Figure 11, the grains visible in the micrograph are divided, with high angle boundaries, and dislocation are dislocations were rarely observed inside the grains. The microstructure is proof of the recrystallisation process, which occurred in the material close to the interface because of the high temperature in this region (see graph in Figure 9). The appearance of dislocation inside the recrystallized grains could have resulted from the load during the upsetting stage of the welding process. The regularly shaped spots visible in the selected area diffraction pattern (Figure 11d) confirm the grain′s lack of significant elastic distortion from the strain induced by the high-density dislocations.

TEM micrographs (Figure 12) show that the intermediate microstructure is visible at a distance of 10 mm from the interface. The reason for this follows from the difference in the microstructure shown in Figure 12a–c. The microstructure (Figure 12a,b), especially Figure 12b, suggests that the recrystallization process occurred in the specimen. On the other hand, the high density of dislocation located around the low-density areas formed dislocation cells that are characteristic of recovery. The difference between the alloy′s microstructure could have resulted from the fact that the observed areas were at the same distance from the interface but located differently with respect to the specimen axis. The electron micrographs (Figure 13) were obtained from thin foil taken at a distance of 20 mm from the interface. Typical dislocations of the cell microstructure are visible in the micrographs (Figure 13a,b). Clearly defined cells, with almost no dislocations inside them were observed. This is a typical microstructure after the recovery process. This microstructure is comparable with that in the starting material, which is usually cold worked (extruded) and then annealed, although the dislocation cells in friction welded specimens are much better defined. Recalling Figure 9, it can see that the temperature at 20 mm from the interface was about 260 °C, which is high enough for the recovery process. However, it should be taken into account that the time of heat exposure was relatively short compared to a conventional heat treatment.

## 5. Conclusions

The following important conclusions were obtained from this study:

(1) The ultimate tensile strength of the joint was comparable to the yield strength of the aluminum alloy, and it was approximately 85% at a friction force of 12.5 kN, friction time of 3.5 s, upsetting force of 50 kN, and upsetting time of 5 s.
(2) Increasing the welding time resulted in a decrease of hardness, mainly when a greater friction force, equal to 22.5 kN, was applied. The local minimum and maximum values were observed as the effect of softening the material in the recrystallisation process. The recrystallisation was caused by maintaining a high temperature for a long time and strain hardening during the upsetting stage of the welding process, the effects of which were not removed by heat-affected processes, e.g., recrystallisation.
(3) The peak temperatures measurements in the axis and at the half-radius of specimens were equal to 252 °C and 278 °C for a distance of 20 mm from the weld interface. The predicted maximum temperatures at the interface were close to 550 °C and 480 °C for the half-radius and at the axis of the aluminum specimens, respectively. The peak temperature was lower than the melting point of aluminum alloy. Moreover, empirical models $T_{max}=550-63.77\cdot x^{0.484},$ and $T_{max}=488-52.64\cdot x^{0.5}$ for estimation of peak temperature were formulated by the authors.
(4) The friction welding process of tungsten composite to aluminum alloy leads to dynamic changes of the aluminum alloy microstructure due to strong plastic deformation. During friction welding of tungsten composite to aluminum alloy recovery and dynamic recrystallisation processes occur. The degree of these processes depends on the temperature and work hardening parameters.

## Figures and Tables

**Table 1:** Alloying elements (wt.%) and the mechanical properties of the base material.

Material	Chemical Composition								Mechanical Properties		
	Al	W	Fe	Ni	Mg	Mn	Si	TS	YS	EL	HB
TC	-	Bal.	2.25	5.25	-	-	-	960	680	27	285
AA	Bal	-	0.194	0.002	2.95	0.265	0.1	276	207	22	83

TS, tensile strength (MPa); YS, yield strength (MPa); EL, elongation (%); HB, hardness.

**Table 2:** Welding parameters used in the friction welding experiments.

Welding Parameters	Values
Friction force (kN)	12.5, 15, 22.5, 25
Friction time (s)	0.5, 3.5, 4.5, 7.5, 9.5
Upsetting force (kN)	50
Upsetting time (s)	5
Rotational speed (rpm)	1450

**Table 3:** Mathematical models for temperature curves during the heating and cooling of the welding process at the half-radius of the specimen.

		Heating Process	
Thermocouple No.	Distance x	Mathematical model $T=\frac{a}{b+\exp\left(c\cdot t\right)}$	*R*-squared $R^{2}$
1	20 mm	$T=\frac{9.409}{0.0303+\exp\left(-0.4104\cdot t\right)}$	0.9978
2	25 mm	$T=\frac{5.226}{0.0209+\exp\left(-0.414\cdot t\right)}$	0.9934
3	30 mm	$T=\frac{9.26}{0.035+\exp\left(-0.347\cdot t\right)}$	0.9914
		Cooling process	
Thermocouple No.	Distance from x	Mathematical model $T=a-b\cdot t^{c}$	*R*-squared $R^{2}$
1	20 mm	$T=-29.11+1.450\cdot t^{-0.5537}$	0.9997
2	25 mm	$T=-192.7+1006\cdot t^{-0.2836}$	0.9974
3	30 mm	$T=-169.8+868.9\cdot t^{-0.2745}$	0.9984

**Table 4:** Mathematical models for temperature curves of the heating and cooling of the welding process in the axis specimen.

		Heating Process	
Thermocouple No.	Distance x	Mathematical model $T=\frac{a}{b+\exp\left(c\cdot t\right)}$	*R*-squared $R^{2}$
1	20 mm	$T=\frac{7.813}{0.0275+\exp\left(-0.3694\cdot t\right)}$	0.9840
2	25 mm	$T=\frac{4.529}{0.0173+\exp\left(-0.3642\cdot t\right)}$	0.9916
3	30 mm	$T=\frac{9.075}{0.0387+\exp\left(-0.2977\cdot t\right)}$	0.9891
		Cooling process	
Thermocouple No.	Distance x	Mathematical model $T=a-b\cdot t^{c}$	*R*-squared $R^{2}$
1	20 mm	$T=-33.97+1501\cdot t^{-0.5579}$	0.9997
2	25 mm	$T=-866.2+1437\cdot t^{-0.0927}$	0.9970
3	30 mm	$T=-446.1+964.3\cdot \;t^{-0.1336}$	0.9916

**Table 5:** Mathematical models for predicted peak temperature for the friction welding process.

Material	Position	Distance x (mm)	Mathematical Model $T\;=\;a-b\cdot x^{c}$	*R*-Squared *R*^2^
AA	half-radius	20, 25, 30	$T=550-63.77\cdot x^{0.484}$	0.9989
AA	axial	20, 25, 30	$T=488-52.64\cdot x^{0.5}$	0.9962

## Data Availability

Not applicable.
